# Trials in adult critical care that show increased mortality of the new intervention: Inevitable or preventable mishaps?

**DOI:** 10.1186/s13613-016-0120-1

**Published:** 2016-02-24

**Authors:** James A. Russell, Mark D. Williams

**Affiliations:** Centre for Heart and Lung Innovation, St. Paul’s Hospital, University of British Columbia, 1081 Burrard Street, Vancouver, BC V6Z 1Y6 Canada; Division of Critical Care Medicine, St. Paul’s Hospital, 1081 Burrard Street, Vancouver, BC V6Z 1Y6 Canada; Indiana University School of Medicine, 1701 North Senate Blvd., Indianapolis, IN 46254 USA

**Keywords:** Mortality, Sepsis, Septic shock, ARDS, Growth hormone, Hetastarch, Nitric oxide synthase inhibitor, Insulin, Oxygen delivery, Adaptive trial design

## Abstract

Several promising therapies assessed in the adult critically ill in large, multicenter randomized controlled trials (RCTs) were associated with significantly increased mortality in the intervention arms. Our hypothesis was that there would be wide ranges in sponsorship (industry or not), type(s) of intervention(s), use of DSMBs, presence of interim analyses and early stopping rules, absolute risk increase (ARI), and whether or not adequate prior proof-of-principle Phase II studies were done of RCTs that found increased mortality rates of the intervention compared to control groups. We reviewed RCTs that showed a statistically significant increased mortality rate in the intervention compared to control group(s). We recorded source of sponsorship, sample sizes, types of interventions, mortality rates, ARI (as well as odds ratios, relative risks and number needed to harm), whether there were pre-specified interim analyses and early stopping rules, and whether or not there were prior proof-of-principle (also known as Phase II) RCTs. Ten RCTs (four industry sponsored) of many interventions (high oxygen delivery, diaspirin cross-linked hemoglobin, growth hormone, methylprednisolone, hetastarch, high-frequency oscillation ventilation, intensive insulin, NOS inhibition, and beta-2 adrenergic agonist, TNF-α receptor) included 19,126 patients and were associated with wide ranges of intervention versus control group mortality rates (25.7–59 %, mean 29.9 vs 17–49 %, mean 25 %, respectively) yielding ARIs of 2.6–29 % (mean 5 %). All but two RCTs had pre-specified interim analyses, and seven RCTs were stopped early. All RCTs were preceded by published proof-of-principle RCT(s), two by the same group. Seven interventions (except diaspirin cross-linked hemoglobin and the NOS inhibitor) were available for use clinically at the time of the pivotal RCT. Common, clinically available interventions used in the critically ill were associated with increased mortality in large, pivotal RCTs even though safety was often addressed by interim analyses and early stopping rules.

## Background

In Critical Care Medicine (CCM), randomized controlled trials (RCTs) are the best evidence to determine whether novel drugs, devices and clinically available therapies are effective [1, 2], safe, superior to “conventional” therapies and recommended for clinical use [3]. We use the term “conventional” to refer to therapies and not to placebo. The Surviving Sepsis Campaign guidelines put a premium on RCTs [3]. “Positive” (i.e., statistically significant) RCTs are sometimes judged as closer to “truth” [2, 4] because some negative RCTs are underpowered.

RCTs must be closely monitored by independent Steering Committees and Data Safety Monitoring Boards (DSMB) complemented by interim analyses and early stopping rules [5]. Superiority design RCTs are done because of prior clinical equipoise, and test the hypothesis that the new intervention is superior to the conventional intervention but use two-sided statistical testing to determine whether either the new or conventional intervention is statistically superior. Thus, new therapy must have clinical equipoise to satisfy ethical concerns [6, 7] and to justify the resources (trial subjects, human staff and financial resources) required. Sometimes, pivotal RCTs were associated with statistically significantly increased mortality with the new intervention [8–13].

Recent reviews consider why so many RCTs in adult sepsis [4] and critical care [2] are not positive, but the subject of excess mortality associated with the intervention group has not been thoroughly reviewed recently. Our hypothesis was that there would be wide ranges in sponsorship (industry or not), type(s) of intervention(s), use of DSMBs, presence of interim analyses and early stopping rules, absolute risk increase (ARI), and whether or not adequate prior proof-of-principle Phase II studies were done of RCTs that found increased mortality rates of the intervention compared to control groups.

## Methods

### RCT selection criteria, inclusion criteria and characteristics of RCTs

We searched published literature for adult critical care RCTs powered for mortality as the primary endpoint that showed statistically significantly increased mortality in the intervention group compared to the control (“conventional”) group(s). We used “trial,” “sepsis,” “critical illness,” “critically ill,” “acute lung injury” and “acute respiratory distress syndrome” key terms. We recorded source of sponsorship, geographical settings, sample sizes, randomization, blinding interventions used, absolute mortality rates, absolute risk increase (ARI), odds ratios, relative risks and number needed to harm, whether or not each RCT had a DSMB, pre-specified interim analyses, whether or not there were pre-specified stopping rules, and whether or not each RCT was stopped early. We also determined whether or not there were prior proof-of-principle RCTs relevant to each RCT and whether the same group did the POP and pivotal RCTs.

## Results

### Characteristics of included RCTs

We found ten adult critical care-related RCTs that were designed and powered for mortality that showed significant increased mortality rates in the intervention compared to the control group(s) (Table 1).

**Table 1:** Subjects, industry sponsorship, geographical settings and interventions tested in adult critical care RCTs with increased mortality in intervention groups

References	Subjects of RCT	Industry sponsored?	Geographical setting	Intervention group	Control group
Hayes [10]	Critically ill	No	UK	High DO2	Normal DO2
Fisher [15]	Septic shock	Yes	USA	TNF-α receptor	Placebo
Sloan [14]	Hemorrhagic shock	Yes	USA	Diaspirin-hemoglobin	Normal saline
Takala [13]	Critically ill	Yes	EU	Growth hormone	Placebo
Edwards [17]	Head injury	No	UK	Methylprednisolone	Placebo
Perner [12]	Severe sepsis	No	Scandinavia	Hetastarch (HES130/0.42)	Ringers lactate
Ferguson [16]	ARDS	No	Canada	High-frequency oscillation	Control ventilation strategy targeting lung recruitment
Finfer [8]	Critically ill	No	Australia/New Zealand/Canada	Intensive insulin	Normal insulin
Lopez [11]	Septic shock	Yes	EU/North America	iNOS inhibitor (546C88)	Placebo
Gao Smith [9]	ALI/ARDS	No	USA	IV salbutamol	Placebo

*DO2* oxygen delivery, *NS* normal saline, *iNOS* inducible nitric oxide synthase, *IV* intravenous, *ARDS* acute respiratory distress syndrome

### Sponsorship and geographical settings

Of the ten RCTs, four were industry sponsored [11, 13–15] and six [8–10, 12, 16, 17] were supported by peer review granting agencies or by academic institutions. The geographical settings were truly global, ranging from single country (USA or Canada only), to regions (Scandinavia), to multicontinent (Asia, EU and North America).

### Conditions studied

There was a wide range of conditions studied including ALI/ARDS [9, 16] (two RCTs), critically ill [8, 10, 13] (three), severe sepsis [12] (one), head injury [17] (one), hemorrhagic shock [14] (one) and septic shock [11, 15] (two).

### Interventions

The RCTs assessed a very wide range of interventions including high oxygen delivery [10], beta-2 adrenergic agonist [9], diaspirin cross-linked hemoglobin [14], human growth hormone [13], methylprednisolone [17], hetastarch [12], high-frequency oscillation ventilation [16], intensive insulin [8], nitric oxide synthase (NOS) [11] inhibitor and TNF-α receptor [15] (Table 1). Remarkably, seven of ten interventions (all except diaspirin cross-linked hemoglobin, the particular NOS inhibitor and TNF-α receptor) were available for use clinically at the time of the pivotal RCT.

### Sample sizes, randomization and blinding

The sample sizes of RCTs also ranged widely from 98 to 10,008 for a total of 19,126 patients (Table 2). Nine of ten RCTs had two groups (intervention and control) that were randomized 1:1. One study was more complex. The trial of human growth hormone [13] was composed of two concurrent RCTs, one a multicenter RCT in Finland and the other a multicenter RCT in many countries; both RCTs randomized patients 1:1 to human growth hormone or placebo.

**Table 2:** Sample sizes, mortality rates, absolute risk increases and number needed to harm (NNH) in adult critical care RCTs showing increased mortality in the intervention groups

References	Sample size Total (intervention/control)	Mortality rates (*n*; %) (intervention/control)	Difference in number of deaths	*P* value	Absolute risk increases (%)	NNH
Hayes [10]	100 (50/50)	27/17 54/34 %	10	0.04	20	5
Fisher [15]	141 (108/33)	59/10 30/48/53/33 %^a^ (45/30)	49	0.02	15	6.6
Sloan [14]	98 (52/46)	24/8 46/17 %	16	0.03	29	3.5
Takala [13]^a^	280 (119/123)	46/24 39/20 %	22	<0.001	19	5.3
Edwards [17]	10,008 (5007/5001)	1248/1075^a^ 25.7/22.3 %	173	0.0001	5	20
Perner [12]	798 (398/400)	203/172 51/43 %	31	0.03	8	12.5
Ferguson [16]	548 (275/273)	129/96 47/35 %	33	0.005	12	8.3
Finfer [8]	6030 (3016/3014)	829/750 27.5/24.9 %	79	0.02	2.6	38.5
Lopez [11]	797 (439/358)	259/175 59/49 %	84	<0.001	10	10
Gao Smith [9]	326 (162/164)	55/38 34/23 %	17	0.02	11	9
Totals or means	19,126 (9626/9462)	2879/2365 29.9/25 %	514		5	20

Four-week mortality rates of albumin versus control groups

In Fisher [15] RCT TNF-α receptor, the primary analysis was a trend test of placebo and increasing doses of TNF-α receptor. Mortality rates are shown for the dose groups and then for the combined intervention versus control groups (45/30)

^a^Six-month mortality rates of methylprednisolone versus placebo

Randomization was blinded in all RCTs. The interventions were blinded in RCTs of diaspirin cross-linked hemoglobin versus normal saline [14], human growth hormone versus placebo [13], methylprednisolone versus placebo [17], hetastarch versus crystalloid [12], nitric oxide synthase (NOS) [11] inhibitor versus placebo, salbutamol versus placebo [9] and TNF-α receptor versus placebo [15].

### Mortality rates, odds ratios, relative risks and absolute risk increases (ARIs)

**Fig. 1:**
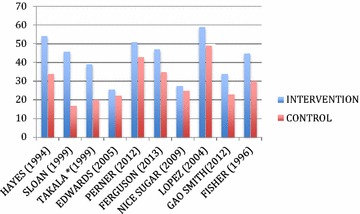
Intervention and control group mortality rates of adult critical care RCTs that found increased mortality of the intervention compared to control groups

**Fig. 2:**
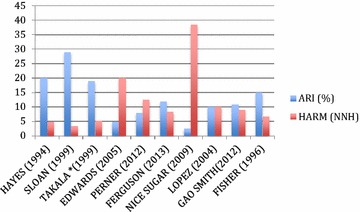
Absolute increased risk of death rates (ARIs) and number needed to harm (NNH) of adult critical care RCTs that found increased mortality of the intervention compared to control group(s)

The primary results showed very wide ranges of intervention and control mortality rates (intervention arms: 25.7–59 %, mean 29.9 %, versus control arms: 17–49 %, mean 25 %) (Table 2; Fig. 1). The number of deaths in intervention groups exceeded the number of deaths in control groups by 514 (Table 2). The odds ratio was reported in only one RCT [8] (odds ratio 1.14 for increased mortality). The relative risks ranged from 1.17 to 2.4 (Table 2). The absolute risk increases (ARIs) ranged widely from 2.6 to 29 % (mean 13 %) such that the number needed to harm also ranged widely from 3.5 to 38.5 (mean 20) (Table 2; Fig. 2).

### Causes of increased mortality: possible off-target effects

Most of the RCTs had secondary findings that might explain the increased mortality rates of the intervention compared to the control groups. Hayes and colleagues [10] assessed increased oxygen delivery; the intervention group had higher doses of dobutamine that were associated with increased risk of tachyarrhythmias—that could have increased mortality. Gao Smith et al. [9] assessed intravenous salbutamol infusion and found more tachyarrhythmias in the intervention than the control arm. NOS inhibition was associated with increased cardiovascular mortality perhaps because of decreased cardiac index [11] in the NOS inhibitor intervention versus control group. Hetastarch causes increased renal toxicity [acute kidney injury (AKI)], and AKI might have increased mortality [18]. HFOV [16] might have decreased venous return, increased the use of vasodilating sedative agents or increased barotraumas compared to control ventilation. Intensive insulin was associated with increased risk of severe hypoglycemia that might explain increased mortality of intensive versus usual insulin [8]. Diaspirin-hemoglobin has vasopressor effects, and the authors speculated that diaspirin-hemoglobin could have increased hemorrhage compared to placebo [14]. However, increased hemorrhage was not documented; we suggest that it is more likely that the vasopressor effects resulted in patients appearing stabilized when they were not. It was hypothesized that removal of TNF-α by TNF-α receptor could have been deleterious [15]. The cause of excess mortality associated with use of human growth hormone was not explained, but the authors speculated mechanisms [13]. There were no “off-target” unexpected complications identified in the primary publications of any of these RCT that could have explained the excess mortality of the intervention arms.

### Interim analyses and early stopping rules

According to the available published information, all but two [10, 15] of the RCTs had independent DSMBs with pre-specified interim analyses and prospectively defined early stopping rules (Table 3). Interestingly, one of the RCTs [10] that had no independent DSMB was the oldest RCT we examined, from an era when interim analyses and early stopping rules were not commonly used. Of note, eight of ten RCTs were stopped early due to potential harm (i.e., increased mortality rate) in the intervention arm (Table 3).

**Table 3:** Frequency and statistical analyses of interim analyses, early stopping and prior Phase II RCTs in adult critical care RCTs showing increased mortality in the intervention groups

References	Interim analyses (frequency)	Interim statistical test	RCT D/C’d early	Prior Phase II RCT (*n*)	DSMB
Hayes [10]	Yes (every 50 patients)	Chi-square	Yes	Yes^e^	No
Fisher [15]	No	NA	No	No	Not stated
Sloan [14]	Yes (after 10, 25, 50 and 75 % enrollment)	Possibly Log rank 28 day^a^	Yes	Yes^f^	Yes
Takala [13]^g^	Yes (group sequential trials; 150 then every 40 patients)	Chi-square	Yes	Yes^c^	Yes
Edwards^d^ [17]	Annually	Chi-square	Yes	Yes$	Yes
Perner [12]	Yes (after 400 patients)	Chi-square	No	Yes	Yes
Ferguson [16]	Yes (pilot phase after 94, 300, 500 and 700 for safety, 800 for efficacy)	Mantel–Haentszel Chi-square	Yes	Yes (*N* = 25)	Yes
Finfer [8]^b^	Yes (after 1500 and 4000 patients)	Chi-square	No	Yes^g^	Yes
Lopez [11]	Yes	Triangular test with Christmas tree correction at stopping boundaries	Yes	Yes (*n* = 312)	Yes
Gao Smith [9]	Yes (every 12 months)	95 % CI	Yes	Yes (*n* = 40)	Yes

Two parallel RCTs in Finland and in Europe **(**UK, the Netherlands, Belgium and Sweden) are reported together [13]. Because of slow recruitment due to the unexpectedly high incidence of exclusion criteria, the design was changed before the first interim analysis. The revised design was a fixed-sample analysis of 170 and 190 patients in the Finnish and multinational studies

*D/C’d* discontinued, *NA* not available in primary publication

^a^The interim analyses methods were not stated. The primary analysis was log rank to 28 days

^b^Prior to the NICE SUGAR RCT [8], van den Berghe et al. [19] had published a similar RCT of intensive versus conventional insulin treatment in critically ill patients

^c^Prior small RCTs in burns, postoperative surgical patients, trauma, sepsis and critically ill non-septic patients

^d^Many prior small RCTs in head injury

^e^Several prior RCTs in septic shock, high-risk surgical, trauma and critically ill patients

^f^Several prior RCTs in trauma and critically ill patients

^g^Several prior trials of intensive insulin

### Prior proof-of-principle RCTs

All but one of the RCTs was preceded by prior proof-of-principle (Phase II) RCTs, and one RCT [8] was preceded by a similar RCT of intensive insulin in the critically ill [19]. One RCT was the first treatment with TNF-α receptor in septic shock [15]. However, according to the primary publication, only two RCTs were preceded by a proof-of-principle RCT by the same group as the pivotal RCT [11, 16].

## Discussion

We found a relatively small number of RCTs in adult CCM that showed that the new intervention was associated with increased mortality compared to control group. Most RCTs were academically sponsored and assessed a wide range of nearly always clinically available interventions. The intervention harm signal was remarkably wide: Absolute risk increase ranged from 2.6 to 29 % (mean 5 %), and the number needed to harm ranged from 3.5 to 38.5 (mean 20). The risks of RCTs finding significantly increased mortality were not explained by the source of sponsorship, geographical settings, lack of blinding, lack of DSMBs or early stopping rules, underlying conditions, inclusion criteria (which ranged widely) or the types of interventions (which also ranged widely). Few prior POP/Phase II RCTs of the particular intervention were done by the same group doing the pivotal/Phase III RCT. While eight of ten RCTs did interim analyses and seven were stopped early, none of the RCTs used response adaptive RCT design.

Ironically these large, pivotal RCTs moved forward because prior proof-of-principle (POP)/Phase II RCTs showed significant results for a surrogate marker chosen to predict success in pivotal Phase III RCTs. Thus, the POP RCTs were false-positive signals of success of pivotal/Phase III RCTs. The reasons for these false-positive signals could include inadequate surrogate markers, changes in dose, changes in usual care, changes in inclusion criteria and settings and/or the play of chance between the POP/Phase II and pivotal/Phase III RCTs. These examples of increased mortality rates of the intervention compared to control groups in pivotal/Phase III RCTs after prior positive POP/Phase II highlight the need for more accurate surrogate markers—biomarker(s) and/or clinical markers—that predict success in pivotal/Phase II RCTs.

We have extended the prior review of increased mortality trials by Freeman et al. [20] who reviewed only human growth hormone, the NO inhibitor L-NMA and diaspirin-hemoglobin. Furthermore, Freeman and colleagues [20] concluded that “factors in the design and conduct of the clinical trial that led to this result be thoroughly discussed.” Ospina-Tascon et al. [21] reviewed methods and quality of multicenter RCTs that had mortality as a primary endpoint and found seven trials as of 2008 that found increased mortality in the intervention group; they concluded that relatively few RCTs “show a beneficial impact of the intervention on the survival of critically ill patients.” We have added to Ospina-Tascon et al. [21] by focusing on RCTs showing harm, and we have updated RCTs reported since 2008. The small number of RCTs in CCM, rigorous Data and Safety Monitoring Board processes, and/or robust proof-of-concept RCTs could explain the low number of harmful RCTs in CCM.

The interventions ranged widely and were nearly always clinically available, suggesting that adult CCM adapted new technologies without positive pivotal RCTs. Clinically available interventions included human growth hormone, methylprednisolone [17], increased oxygen delivery [10], novel resuscitation fluid for resuscitation [12], high-frequency oscillation ventilation [16] and salbutamol. There were three interventions that were not available clinically: diaspirin cross-linked hemoglobin [14], NO synthase (iNOS) inhibitor L-NMA [11] and TNF-α receptor [15].

An alternative interpretation is that the new interventions were unproven, that only large pivotal Phase III RCTs powered for mortality would prove efficacy, and that inevitably some interventions increase mortality in Phase III. Thus, some argue such RCTs are necessary—indeed critical—to guide clinical practice away from harmful interventions.

Finding increased mortality of the “conventional” group is essentially showing that the newer therapy is more effective and that is the goal of the vast majority of superiority design RCTs in critical care. We chose to search for and review papers in which the results were the opposite of the initial hypothesis—i.e., the “conventional” intervention was superior, and the new therapy significantly increased mortality. Our contention is the same regardless of design, i.e., we aim to minimize harm from new interventions in future RCTs. If more patients die with the conventional therapy, that is the same as showing that the new therapy is more effective. We thus believe it does matter whether the increased mortality is in the “conventional” therapy group (i.e., showing more benefit with the new intervention) versus in the new therapy group (i.e., the hypothesis that the new intervention was better is not merely not shown, and the exact opposite to the hypothesis is shown). We did not avoid RCTs in which there are contrasting strategies in which none can be considered as “new” or “conventional.”

Equipoise is a crucial issue for our study of RCTs showing increased mortality with the new intervention. One might consider that if there was equipoise regarding interventions prior to RCTs (and there must be equipoise for ethical conduct and ethics approval of RCTs), then one might suppose that there would have been a similar number of RCTs showing increased mortality with the new intervention as the number of RCTs showing increased mortality with the “conventional” intervention. It is possible that RCTs that showed increased mortality with the “conventional” intervention are much more numerous than the inverse; however, the low number of RCTs in CCM—sepsis in particular—that found significantly lower mortality with the new intervention suggests that it is not the case that RCTs that showed increased mortality with the “conventional” intervention are much more numerous than the inverse.

It is also possible that harmful effects of interventions are more easily identified than beneficial effects, for example, in a subgroup. In a hypothetical placebo-controlled study of penicillin in sepsis, where beneficial effects in a small subgroup of patients would be difficult to prove, anaphylactic reactions would be well recognized. This heterogeneity of treatment effect in detection of benefit versus harm was reviewed elegantly in simulations of sepsis RCTs by Iwashyna et al. [22]. They show that positive RCTs (i.e., beneficial overall) could have buried in them subgroup(s) with consistent harm or little benefit such as low-risk patients who met enrollment criteria.

It is possible that some RCTs that found increased mortality with the “new intervention” were not published (especially before clinicaltrials.gov registration). If the following supposition were true—there were the same number of RCTs in which both new and old intervention increased mortality—then that supposition would indicate that the vast majority of RCTs were unable to show anything other than equivalence, or non-inferiority.

Response adaptive trial design might decrease the risk of RCTs that show increased mortality of intervention versus control group(s). Response adaptive trial design RCTs adjust group randomization (intervention or control) and sometimes dose while the RCT progresses by using ongoing interim results. This is scientifically sound when the algorithms for group/dose assignment are comprehensively pre-specified and investigators and sponsors cannot adjust ongoing randomization. Strengths of response adaptive trial design include more efficient assessment of efficacy, limited risk of RCTs that find potential harm (by decreasing the sample size compared to frequentist statistical trial design), decreased time and expense (i.e., more efficient futility rules), improved drug dose selection and earlier completion of negative RCTs. We do acknowledge that this approach could limit the enrollment of a sufficient number of patients to be highly confident about mortality differences.

Response adaptive trial design often adjusts sample size to prevent an underpowered RCTs or excessively large RCTs when the treatment effect is larger than expected. Interim analyses of PROWESS-SHOCK lead to increased sample size because of lower than expected blinded mortality [23, 24]. Response adaptive trial designs are now used in proof-of-concept RCTs in critical care (e.g., l-carnitine [25]) and pivotal RCT of selepressin versus placebo in septic shock (https://clinicaltrials.gov/ct2/show/NCT02508649?term=selepressin&rank=1). The regulatory bodies (FDA and EMEA) have accepted this design recently [4] (https://clinicaltrials.gov/ct2/show/NCT02508649?term=selepressin&rank=1).

Patient safety in RCTs necessitates methods to decrease the risk of excess deaths due to new interventions [26]. Response adaptive trial design could decrease the risk of increased mortality only after a large sample size has been evaluated (27) by adjusting randomization to the intervention or control as the RCT progresses based on ongoing interim analyses. Some believe that Phase III mortality RCTs that stop early for efficacy overestimate treatment efficacy [27].

Most RCTs had secondary results that might explain higher mortality rates of intervention compared to control groups such as increased cardiovascular complications (tachyarrhythmias possibly caused by the interventions in two RCTs (dobutamine [10]; intravenous salbutamol infusion [9]), decreased cardiac output by excessive vasoconstriction due to NOS inhibition [11] or by decreased venous return secondary to high-frequency oscillation ventilation [16]), renal toxicity (hetastarch increased risk of acute kidney injury [12, 18]) and hypoglycemia (intensive insulin had significantly increased severe hypoglycemia [8]). The causes of excess mortality associated with human growth hormone and methylprednisolone [17] were not explained although speculations were presented [13]. Removal of TNF-α by TNF-α receptor was hypothesized to be potentially deleterious [15]. Larger Phase II proof-of-concept RCTs could have clarified safety risks thus modifying (e.g., drug dose, duration, specific safety signal monitoring) or avoiding pivotal Phase III RCTs.

All but one of the RCTs was preceded by prior proof-of-principle Phase II RCTs, and another prior pivotal Phase III [8] RCT by the same group preceded the pivotal RCT. We speculate that larger, dose response adaptive trial design of Phase II RCTs in CCM could limit safety risk and increase the probability of technical success of Phase III RCTs.

Cardiology has improved outcomes through well-designed, large RCTs and emphasizes a model of cooperation between academia and industry [28]. Cardiology RCTs adjusted design from lessons learned from earlier missteps. For example, the CAST trial [29, 30] enrolled patients at risk of ventricular arrhythmias and randomized to the anti-arrhythmics moricizine, encainide or flecainide versus placebo. At the first interim analysis, the DSMB recommended stopping the encainide and flecainide arms (pooled mortality was higher than placebo). Ironically, a later editorial bemoaned that two “potentially efficacious” drugs could be removed from clinical usage, not mentioning the increased mortality [30]. CAST catalyzed rigorous, independent monitoring of RCTs [31] [e.g., Academic Research Organizations in independent DSMBs and regulatory guidance on DSMB function (www.fda.gov/OHRMS/DOCKETS/98fr/01d-0489-gdl0003.pdf)].

Our review of RCTs that found increased mortality in the intervention arm in CCM emphasizes caution in the design and monitoring of future RCTs in critically ill patients. Our review of Phase II proof-of-principle RCTs aligns with recent emphasis on Phase II RCTs [32] and complements other suggestions to improve chances of success in critical care RCTs [4].

The strengths of our analyses are wide inclusion criteria, careful screening of the adult CCM RCT literature, detailed evaluation of many aspects of RCT design and implementation and consideration of RCT- and intervention-specific risks—as opposed to design risks. Shortcomings are that we did not have access to original RCT data to model how the use of response adaptive trial design could have decreased risk of excessive intervention group mortality rates, we did not search for secondary publications that could have explained the causes of increased mortality in the intervention groups, and our findings may not apply to other fields (critically ill patients have increased risk of adverse events, have a high mortality and receive numerous interventions).

## Conclusions

Some common, clinically available interventions used in critically ill patients increased mortality in large multicenter pivotal Phase III RCTs in adult critical care. We found wide ranges in sponsorship (industry or not), type(s) of intervention(s), use of DSMBs, presence of interim analyses and early stopping rules, absolute risk increase (ARI), and whether or not adequate prior proof-of-principle Phase II studies were done of RCTs that found increased mortality rates of the intervention compared to control groups. We suggest new approaches to decrease risk of harm in pivotal Phase III RCTs in the critically ill including better surrogate endpoints in POP/Phase II RCTs to more accurately predict success in pivotal/Phase III RCTs, larger proof-of-principle/Phase II RCTs and use of response adaptive trial design in Phases II and III.
